# Prevalence and geographic distribution of *Echinococcus* genus in wild canids in southern Québec, Canada

**DOI:** 10.1371/journal.pone.0306600

**Published:** 2024-07-15

**Authors:** Ève-Marie Lavallée-Bourget, Christopher Fernandez-Prada, Ariane Massé, Patricia Turgeon, Julie Arsenault

**Affiliations:** 1 Department of Pathology and Microbiology, Faculty of Veterinary Medicine, Université de Montréal, Saint-Hyacinthe, Québec, Canada; 2 Epidemiology of Zoonoses and Public Health Research Unit (GREZOSP), Faculty of Veterinary Medicine, Université de Montréal, Saint-Hyacinthe, Québec, Canada; 3 Ministère de l’Environnement, de la Lutte Contre les Changements Climatiques, de la Faune et des Parcs, Québec, Québec, Canada; 4 Public Health Agency of Canada, Saint-Hyacinthe, Québec, Canada; George Washington University School of Medicine and Health Sciences, UNITED STATES

## Abstract

*Echinococcus* spp. is an emerging zoonotic parasite of high concern. In Canada, an increase in the number of human and animal cases diagnosed has been reported, but information regarding the parasite’s distribution in wildlife reservoir remains limited. A cross-sectional study was conducted to estimate the prevalence of wild canids infected with *Echinococcus* spp. and *Echinococcus multilocularis* in areas surrounding populated zones in Québec (Canada); to investigate the presence of areas at higher risk of infection; to evaluate potential risk factors of the infection; and as a secondary objective, to compare coproscopy and RT-PCR diagnostic tests for *Taenia* spp. and *Echinococcus* identification. From October 2020 to March 2021, fecal samples were collected from 423 coyotes (*Canis latrans*) and 284 red foxes (*Vulpes vulpes*) trapped in 12 administrative regions. Real-time PCR for molecular detection of genus *Echinococcus* spp. and species-specific *Echinococcus multilocularis* were performed. A total of 38 positive cases of *Echinococcus* spp., of which 25 were identified as *E*. *multilocularis*, were detected. Two high-risk areas of infection were identified. The prevalence of *Echinococcus* spp. was 22.7% (95% CI 11.5–37.8%) in the Montérégie centered high-risk area, 26.5% (95% CI 12.9–44.4%) in the Bas-St-Laurent high-risk area, and 3.0% (95%CI 1.8–4.7%) outside those areas. For *E*. *multilocularis*, a prevalence of 20.5% (95% CI 9.8–35.3%) was estimated in the high-risk area centered in Montérégie compared to 2.4% (95% CI 1.4–3.9%) outside. Logistic regression did not show any association of infection status with species, sex, or geolocation of capture (*p* > 0.05). This study shows the circulation of *Echinococcus* in a wildlife cycle in 9/12 administrative regions of Québec.

## Author summary

*Echinococcus* spp., including *E*. *multilocularis*, are zoonotic parasites spread by wild canids that exhibit spatial expansion and emergence in North America. Given their ability to cause severe lesions and mortality in the absence of treatment, the recent detection of the first human case of *E*. *multilocularis* in Québec in 2018 raised significant public health concerns. In this study, we estimated the prevalence and described the spatial distribution of infection with parasites of the genus *Echinococcus* in coyotes (*Canis latrans*) and red foxes (*Vulpes vulpes*) near populated areas in southern Québec. A total of 707 carcasses of coyotes and red foxes were collected from 12 administrative regions. Fecal samples from each carcass were analyzed using microscopy-based and molecular methods. We detected *Echinococcus* spp. and *E*. *multilocularis* in 8/12 of the studied areas. We found two high-risk areas of infection. Our results demonstrate that *Echinococcus* spp. is circulating in Québec and highlight the importance of implementing preventive measures to limit human and domestic animal infections and of raising awareness among at risk animal populations.

## Introduction

Echinococcosis is a zoonotic worldwide-distributed disease mainly caused by two cestode species, *Echinoccoccus granulosus (sensu lato)* and *Echinococcus multilocularis* [1]. In Canadian wildlife, two *Echinococcus* species are endemic: *E*. *canadensis* (*E*. *granulosus sensu lato* genotypes 8 and 10) and *E*. *multilocularis* [2]. They respectively cause cystic and alveolar echinococcosis, two potentially fatal diseases in humans when left untreated [1, 3]. Despite advancements in diagnosis and treatments of these conditions, their impact on human and animal health remains significant worldwide [4]. In Canada, an average cost of $8 842 CAD per case was estimated for the treatment of human cystic echinococcosis [5]. Humans are considered accidental hosts and they can acquire the infection by inadvertent ingestion of the parasite eggs either contaminating fresh food, water, soil, or adhered to the fur of definitive hosts [2, 6]. After ingestion by an intermediate host or a human, the eggs will hatch into larvae in the intestines, which will penetrate the intestinal wall and migrate to one of the target organs, mainly the liver or the lungs [1]. In humans, the cystic lesion will develop following a long incubation period of 5 to 15 years [7]. Domestic dogs and other canids act as definitive hosts by cyst ingestion. Unlike *Taenias*, adult worms of *Echinococcus* are only a few millimeters long, but the eggs of the two genera are very similar [8]. Dogs could also develop the disease as aberrant intermediate hosts with accidental egg ingestion. As the diagnosis is often late in dogs as aberrant intermediate hosts, the prognosis of echinococcosis is frequently reserved despite the treatments implemented [9].

Both *Echinococcus* species found in Canada circulate in a sylvatic cycle involving wild canids ((wolves (*Canis lupus*), coyotes (*Canis latran*s), red foxes (*Vulpes vulpes*) and arctic foxes (*Vulpes lagopus*)) or domestic dogs (*Canis canis*), as definitive hosts [10, 11]. In the province of Québec, moose (*Alces alces*), white-tailed deer (*Odocoileus virginianus*) and caribou (*Rangifer tarandus*) [12] are the known intermediate hosts needed to complete the parasite cycle for *E*. *canadensis*. This species was found in wildlife in all Canadian provinces, except for the northernmost Arctic islands and the Atlantic provinces [11]. For *E*. *multilocularis*, known intermediate hosts in Québec are rodent species including meadow vole (*Microtus pennsylvanicus*), red-backed vole (*Myodes gapperi*) and deer mouse (*Peromyscus maniculatus*) [13]. In Canada, *E*. *multilocularis* distribution was historically limited in the northern region [14, 15], but recent findings revealed an expansion of the parasite’s geographical range in several provinces which are now considered endemic [7, 16]. However, *E*. *multilocularis* was neither found in the province of Québec in wolves, coyotes or red foxes based on a study conducted in 2016–17 [10]. In the southern part of the bordering province of Ontario, the prevalence in wild canids during the same period was estimated at 23% (95% CI = 20–27%) [7]. Moreover, since 2012, five cases of alveolar echinococcosis have been described in dogs in Ontario [7].

Wild canids pose a significant risk of environmental contamination and parasite dispersal [17]. Coyotes have large home range size varying from 7 to 80 km^2^ on average and transient animals can travel more than 300 km away from their den [18–21], which is likely to increase the risk of environmental contamination and egg dispersal [18, 22–24]. Pre-patent period, defined as the period between infection in the definitive host and fecal shedding of eggs, is suspected to be as short as 26 to 29 days based on studies done on hamsters and red foxes for *E*. *mutlilocularis* and excretion persists for about 4 months [3, 25]. The number of adult worms in the intestine ranges from 5 to 6038 per dog (median of 1508) and each worm can produce thousands of eggs every day [10, 25, 26]. Worm burden was estimated to be between 40–12 200 for red foxes and between 260–91144 in coyotes in a study done in Alberta, Canada [27]. Variations in worm burden in definitive hosts indicates that a small part of animal harbor most of worm burden [28]. Eggs are resistant to heat and can remain infective for months under optimal conditions, which makes environmental decontamination difficult [3]. Since the last century, coyotes have expanded their range in Canada and the United States, and are recently increasingly found in urban areas, attracted by easily accessible food [18, 29, 30]. In the census metropolitan area of Calgary, *E*. *multilocularis* was identified in 20.5% of coyotes sampled [31]. This information raises questions about the impact of this zoonotic parasite because its detection in urban and agricultural settings highlights the risk of exposure of humans and domestic animals to environmental contamination brought by coyotes [23, 31].

The emergence of *Echinococcus* with the actual global urbanization is of growing concern secondary to the geographic expansion of definitive hosts, identification of new *Echinococcus* strains and detection of new endemic areas [2, 7, 18, 31–33]. In Québec, the first human case of alveolar echinococcosis was diagnosed in 2018 in a young child from the Laurentides region, suggesting that the spatial distribution of the parasite is overriding provincial borders [34]. Therefore, a better understanding of the prevalence among coyotes and red foxes across the province could help in assessing the exposure risk in human and domestic dogs and in orienting prevention strategies at a regional level. This research project aims to (i) estimate the prevalence of *Echinococcus* spp. and *E*. *multilocularis* infection in coyotes and red foxes in populated areas in Québec, (ii) identify high-risk areas of infection and, (iii) investigate the potential risk factors of the infection. As a secondary objective, the probability of detecting *Echinococcus* spp. and *E*. *multilocularis* by real-time PCR (RT-PCR) was compared between fecal samples with and without *Taenia* spp. eggs, from a surveillance perspective.

## Methods

### Ethics statement

The study protocol was approved by the Animal Use Ethics Committee of the Faculty of Veterinary Medicine at Université de Montréal (CÉUA #22-Rech-2181).).

### Study area and animal collection

The study was conducted in the inhabited area of 12 administrative regions (out of 17) of the province of Québec, Canada (Outaouais, Laurentides, Lanaudière, Montréal, Laval, Montérégie, Estrie, Mauricie, Centre-du-Québec, Capitale-Nationale, Chaudière-Appalaches and the Bas-St-Laurent). These regions were selected as they form a geographically continuous area encompassing the largest cities and most populated regions of the province. A target sample size of 50 coyotes per region was determined to enable the detection of the parasite in each region with a 95% confidence level assuming a prevalence of at least 6% in the population. Red foxes were then conveniently added up to the maximum possible given our resources.

In each region, trappers were informed of the project through their regional trapper associations. Those interested were contacted by the research team and recruited on a voluntary basis. From October 2020 to March 2021, participating trappers provided coyote and red fox carcasses harvested as part of their regular annual fur collection and/or pest control activities with full compliance of provincial trapping regulations. The trappers were informed that only animals trapped in peri-urban or agricultural areas, excluding forested areas, were eligible for the study. Within each region, the numbers of trapped coyotes and red foxes to be provided were distributed among recruited trappers proportionally to the number they had trapped in previous years. For each trapper, the first carcasses were selected until their attributed number was reached. A $35 CAD was offered for each carcass submitted as compensation for the extra time needed to collect organs, record information, and prepare shipments. Additional carcasses collected in Montréal-Laval, Estrie and Montérégie by the regional offices of the Québec *Ministère de l’Environnement*, *de la Lutte aux changements climatiques*, *de la Faune et des Parcs* (MELCCFP; Ministry of the Environment, Climate Change, Wildlife and Parks) following road accidents or found dead and reported by the citizens were also included in our study.

Participating trappers received a detailed protocol for data collection, and organ sampling, as well as the necessary material for sampling and shipment. For each carcass, the sex, species and capture location (geographical coordinates, projected coordinates, or municipality of the capture site) were recorded by trappers. Thoracic and abdominal viscera were removed from the carcasses by trappers, packed and kept at -20˚C until shipment to the Faculty of Veterinary Medicine in Saint-Hyacinthe, Canada. Upon receipt, viscera were frozen for a minimum of 4 days at -80˚C to inactivate *Echinococcus* eggs and protect staff from exposure [32]. Subsequently, they were slowly thawed (4˚C) for 48–72 hours. Thoracic and abdominal viscera were examined for macroscopic lesions compatible with *Echinococcus* by visual inspection and palpation. If present, these lesions were collected and fixed in formalin, stained with hematoxylin and eosin (H&E) and submitted for histopathological examination. Intestinal content from duodenum to colon was collected by manual pressure and mixed in a small container. After homogenization, one aliquot of fecal material was stored at 4˚C for a maximum of two days prior to coproscopy analysis. Another aliquot was stored at -80˚C for a maximum of 96 days before RT-PCR testing.

### Laboratory analyses

Microscopy-based coproanalyses were performed at the AVVLD-accredited Animal Parasitology Diagnostic Laboratory of *Centre de Diagnostic Vétérinaire de l’Université de Montréal* (CDVUM) in St-Hyacinthe (Canada). Briefly, 2.0 ± 0.1 g of fecal material were added to 10 mL of ZnSO_4_ and centrifuged at 1.050 × g for 2 min. Following centrifugation, 2 mL of fluid from the meniscus was removed and washed three times in 1 × PBS pH 7.4 (with centrifugation at 1.650 × g for 5 min after each wash), and the pellet was resuspended to a final volume of 1 mL of 1 × PBS. Parasitic elements (including *Taenia* spp. eggs) were identified and counted by a certified parasitology technician. Eggs of *Echinococcus* cannot be differentiated at the genus level from other taeniid eggs under microscope based on morphology, the presence of *Taenia* spp. eggs was noted and the sample further tested by molecular biology to identify true positives [35].

All fecal samples were tested for the presence of *Echinococcus* by RT-PCR using the IDEXX Echinococcus RealPCR™ (IDEXX, California) following manufacturer instructions. The DNA extraction and RT-PCR protocol is fully described in a previous study [36]. Initially, a RT-PCR assay for *Echinococcus* spp. detection targeting a mitochondrial rRNA single-copy gene located in a region between COX1 and tRNA-THR genes was performed. RT-PCR was run with seven quality controls including PCR positive controls, PCR negative controls, negative extraction controls, DNA pre-analytical quality control targeting the host SSRs (18S rRNA) gene complex, an internal positive control spiked into the lysis solution, and an environmental contamination monitoring control. RT-PCR was considered positive when Ct value was lower than 40. In case of positive RT-PCR for *Echinococcus* spp., a second RT-PCR targeting a single copy mitochondrial rRNA gene specific for *E*. *multilocularis* detection was executed with the same protocol. These assays were designed with an analytical sensitivity to detect at least 1–10 genome equivalents of the pathogens with an analytical specificity of above 95%. The clinical sensitivity and specificity of these RT-PCR tests were not available.

### Spatial cluster detection

The geographic distribution of sampled coyotes and red foxes was mapped according to their RT-PCR status using ArcGIS Pro 3.1.1 (Environmental Systems Research Institute, Redlands, CA, USA). For mapping, each carcass was geocoded at trap site using the coordinates (latitude, longitude) provided by trappers. In 330 samples, when coordinates were missing, the carcass was georeferenced at the centroid of the municipality of capture according to the online Google Maps^®^ application. All coordinates were then converted to Lambert conformal conic NAD83 projection system prior to mapping. We used reference maps from Statistics Canada [37]. The study area was illustrated using the population ecumene of the selected administrative regions from the 2016 census of Statistics Canada.

We used the Kulldorff spatial scan statistic with the Bernouilli model for the detection of spatial clusters of positivity for *Echinococcus* spp. or *E*. *multilocularis* with SaTScan^TM^ software, version 9.6 [38]. The test was carried out independently for *Echinococcus* spp. and *E*. *multilocularis*, by animal species and for all canids combined. The maximum cluster size was set at 50% of the total sample size. Statistical significance of spatial clusters was determined through 9999 Monte Carlo replicates using an alpha of 0.05 for interpretation.

We performed statistical analyses with SAS^®^ software version 9.4 (SAS Institute, Cary, N.C., USA). Prevalence of RT-PCR-positive *Echinococcus* spp. and *E*. *multilocularis* in coyotes and red foxes with 95% Clopper-Pearson exact confidence intervals were estimated by administrative regions. In the absence of evidence of regional or animal species differences in the risk of infection, as informed by the risk factor and spatial cluster analyses, we estimated the overall prevalence with an exact 95% Clopper-Pearson confidence interval.

### Risk factors analysis

Descriptive statistics were used to report the characteristics of animals according to their status for *Echinococcus* spp. and *E*. *multilocularis*. Two logistic regression models were developed using the *Echinococcus* spp. or *E*. *multilocularis* infection status (RT-PCR positive or negative) as outcomes. Species (coyote vs. red fox), sex (male vs. female), projected X-coordinates (continuous) and projected Y-coordinates (continuous) and inclusion in a spatial cluster (yes or no) were used as explanatory variables. The latter variable was used to account for potential unmeasured environmental confounders. Projected coordinates were scaled and centered on their mean. The linearity of the relationships between the predicted logit and projected coordinates were evaluated graphically using univariable logistic regression after categorizing projected coordinates into 3 equal-interval categories. All explanatory variables were first included in full models. No adjustment for trappers was included, as clustering by trapper was not expected given that red foxes generally live alone during autumn and winter before the breeding season, and coyotes also live alone or in small groups of 2, 3 or 4 individuals [19]. Also, coyotes and red foxes have large home ranges, which extend respectively from 7–80 km^2^ and 0.95–44 km^2^ [7, 19, 39–41]. A backward procedure was then applied to build the final models, by sequentially removing explanatory variables using a criterion of p > 0.05 (likelihood ratio test) for removal. The Homer-Lemeshow test was used to assess the fit of the final models. Finally, the risk ratio with 95% confidence limits was used to compare the probability of RT-PCR positivity according to taeniid eggs detection.

## Results

### Laboratory results

From October 29^th^, 2020 to March 8^th^, 2021, 702 carcasses were submitted by 57 trappers and 5 carcasses by the MELCCFP, for a total of 707 carcasses. Among carcasses, 59.8% were coyotes (n = 423) and 40.2% were red foxes (n = 284). No samples were obtained in the Laval administrative region. Overall, 38 fecal samples (24 coyotes, 14 red foxes) were RT-PCR-positive for DNA detection of *Echinococcus* spp. and among them, 25 (14 coyotes, 11 red foxes) were also RT-PCR-positive for *E*. *multilocularis*. Ct values ranged between 21 to 37 for *Echinococcus* spp. and between 22 to 40 for *E*. *multilocularis* (S1 and S2 Figs).

We noted macroscopic lesions suggestive of parasitic cysts on the liver of 4 red foxes and the lungs of 1 coyote. None of these were compatible with *Echinococcus* infection according to histopathologic examination and were also negative to RT-PCR.

### Prevalence of infection

**Fig 1:**
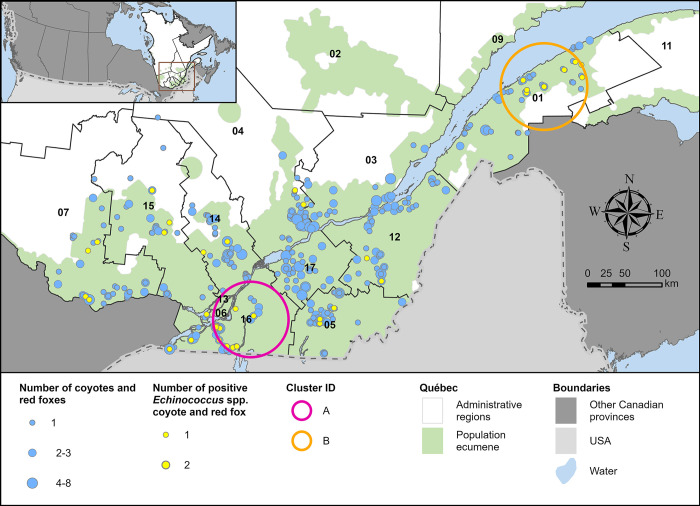
Distribution of 707 coyotes (n = 423) and red foxes (n = 284) according to their RT-PCR status for *Echinococcus* spp. in Québec (Canada), 2020–2021. Spatial clusters of infection described in Table 1 are represented. Numbers identify administrative regions (01 –Bas-St-Laurent, 02 –Saguenay-Lac-St-Jean, 03 –Capitale-Nationale, 04 –Mauricie, 05 –Estrie, 06 –Montréal, 07 –Outaouais, 09 –Côte-Nord, 11 –Gaspésie-Îles-de-la-Madeleine, 12 –Chaudières-Appalaches, 13 –Laval, 14 –Lanaudière, 15 –Laurentides, 16 –Montérégie, 17 –Centre-du-Québec).

**Fig 2:**
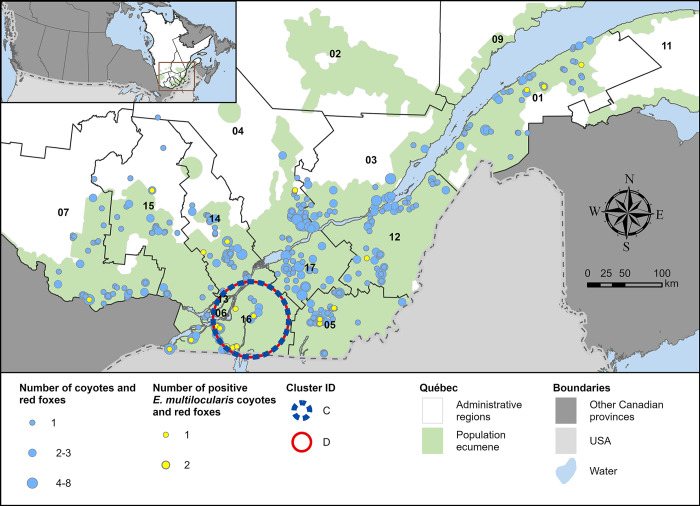
Distribution of 707 coyotes (n = 423) and red foxes (n = 284) according to their RT-PCR status for *E*. *multilocularis* in Québec (Canada), 2020–2021. Spatial clusters of infection described in Table 1 are represented. Numbers identify administrative regions (01 –Bas-St-Laurent, 02 –Saguenay-Lac-St-Jean, 03 –Capitale-Nationale, 04 –Mauricie, 05 –Estrie, 06 –Montréal, 07 –Outaouais, 09 –Côte-Nord, 11 –Gaspésie-Îles-de-la-Madeleine, 12 –Chaudières-Appalaches, 13 –Laval, 14 –Lanaudière, 15 –Laurentides, 16 –Montérégie, 17 –Centre-du-Québec).

*Echinococcus* spp. was detected by RT-PCR in 9/12 of administrative regions under study, while no positive case was observed in Capitale-Nationale and Centre-du-Québec (Fig 1, S1 and S2 Tables). In areas where the parasite was identified, the minimum and maximum regional prevalence in coyotes was 2.3% in Lanaudière and 20.0% in Montréal, and in red foxes, it was 2.3% in Lanaudière and 15.4% in Montérégie. *E*. *multilocularis* was detected in 7/12 of the studied regions as no canid tested positive in Capitale-Nationale, Centre-du-Québec and Montréal (Fig 2, S1 and S2 Tables). In the administrative regions where *E*. *multilocularis* was detected, the minimum and maximum regional prevalence was 1.9% in Chaudières-Appalaches and 16.7% in Montérégie for coyotes, and in red foxes it was 2.3% in Lanaudière and 12.8% in Montérégie. In this study, 2 coyotes were collected on the same site and day by the same trapper and were RT-PCR positive for *Echinococcus* spp. and *E*. *multilocularis*, which might represent individuals from the same pack sharing the same infection risk. Other RT-PCR positive intestinal content from carcasses were collected at a different time or a different location by a different trapper, supporting our assumption of independence for statistical analyses.

### Spatial clusters of infection

Two high-risk areas of infection were detected (represented in Figs 1 and 2), one centered in Montérégie and the other one centered in Bas-St-Laurent associated with four significant clusters covering these zones (described in Table 1).

**Table 1:** Characteristics of the four spatial clusters of infection of *Echinococcus* spp. and *E*. *multilocularis* in coyotes (C, n = 423) and red foxes (R, n = 284) in Québec (Canada) 2020–2021.

Cluster ID	A	B	C	D
**Centroid of the high-risk area**	Montérégie	Bas-St-Laurent	Montérégie	Montérégie
**Outcome**	*Echinococcus* spp.	*Echinococcus* spp.	*E*. *multilocularis*	*E*. *multilocularis*
**Animal species**	C + R	C + R	C	C + R
**Radius of the high-risk area (km)**	51	58	51	51
**No. of animals in the high-risk area**	44	34	21 C	44
	(21 C + 23 R)	(22 C + 12 R)		(21 C +23 R)
**No. of cases in the high-risk area**	10	9	6 C	9
	(6 C + 4 R)	(6 C + 3 R)		(6 C + 3 R)
**Relative risk (%) (95% CI)**	5.4 (2.8–10.4)	6.1 (3.2–11.9)	14.4 (5.5–37.6)	8.5 (4.0–18.1)
**Prevalence inside high-risk area (%) (95% exact CI)**	22.7 (11.5–37.8)	26.5 (12.9–44.4)	28.6 (11.3–52.2)	20.5 (9.8–35.3)
**Prevalence outside high-risk area (%) (95% exact CI)**	3.0 (1.8–4.7)		2.0 (0.9–3.9)	2.4 (1.4–3.9)

For *Echinococcus* spp., we did not observe any significant cluster of infection by considering the animal species separately. Significant clusters were centered in Montérégie (cluster A) and Bas-St-Laurent (cluster B) (Fig 1, Table 1).

For *E*. *multilocularis*, two significant clusters of infection were observed with both their centroid located in Montérégie and covering the same geographic area (Fig 2). One included coyotes only (cluster C) whereas the other included both species (cluster D) (Table 1).

### Risk factors

The logit of the two continuous predictors (projected coordinates) was not linear according to visual assessment, and these two variables were recoded into 3 equal interval categories for inclusion in the model. The proportion of positive canids according to animal species, sex, geographic coordinates and inclusion in high-risk areas are shown in Table 2. Except for the inclusion in high-risk areas, none of the explanatory variables were significantly associated with *Echinococcus* spp. or *E*. *multilocularis* according to the final logistic regression models (Table 2), as all variables were removed from the model (p-value > 0.05) during the selection procedure.

**Table 2:** Descriptive statistics and p-values from full logistic regression models predicting RT-PCR positivity for *Echinococcus* spp. or for *E*. *multilocularis* according to species, sex, projected coordinates and inclusion in high-risk area in wild canids collected in Québec, Canada (2020–2021).

Characteristics	No tested	*Echinococcus* spp.			*E*. *multilocularis*		
		No of positives	% of positives	p-value^1^	No of positives	% of positives	p-value^1^
**Species**				0.326			0.889
Coyotes	423	24	5.7		14	3.3	
Red foxes	284	14	4.9		11	3.9	
**Sex** ^**a**^				0.832			0.861
Male	350	21	3.1		14	2.1	
Female	320	15	2.2		10	1.5	
**Projected X-coordinates**				0.085			0.551
-62.40 − -38.76	237	18	7.6		12	5.1	
-38.53 − -15.16	379	11	2.9		9	2.4	
-14.73 − 9.08	91	9	9.9		4	4.4	
**Projected Y-coordinates** ^**b**^				0.763			0.398
12.58 − 26.40	381	22	5.8		17	4.5	
26.71 − 40.57	253	7	2.8		4	1.6	
42.12 − 54.68	72	9	12.5		4	5.6	
**Inclusion in high-risk area**				< 0.001			<0.001
No	629^c^; 663^d^	19	3.0		16	2.4	
Yes	78^c^; 44^d^	19	24.3		9	20.5	

^a^ Sex was missing in 37 animal samples

^b^ Projected Y-coordinate was missing in one sample

^c^ For *Echinococcus* spp. models

^d^ For *E*. *multilocularis* models

^1^ According to the likelihood ratio test

### Association between coproscopy and real-time PCR detection

We detected eggs of *Taenia* spp. in 57 fecal samples (8.06%; 46 coyotes and 11 red foxes) based on microscopic morphologic features. From the 57 fecal samples positive for *Taenia* spp. at coproscopy, 22.81% and 14.04% were also RT-PCR positive for *Echinococcus* spp. and for *E*. *multilocularis*, respectively (Table 3). The risk of RT-PCR positivity was 5.93 (95% CI = 3.21–10.95) times higher for *Echinococcus* spp. and 5.37 (95% CI = 2.42–11.89) time higher for *E*. *multilocularis* in fecal samples with eggs of *Taenia* spp. eggs detected compared with negative samples.

**Table 3:** Comparison of coproscopy identification of *Taenia* spp. eggs and RT-PCR detection of *Echinococcus* spp. or *E*. *multilocularis* in coyotes and red foxes (n = 707).

*Taenia* spp. eggs detection in coproscopy	Number of animals	Number (%) of positive RT-PCR animals	
		*Echinococcus* spp.	*E*. *multilocularis*
**Positive**	57	13 (22.81%)	8 (14.04%)
**Negative**	650	25 (3.85%)	17 (2.62%)

## Discussion

Our results demonstrate the widespread distribution of *Echinococcus* spp. in Québec and represent the first report of the circulation of *E*. *multilocularis* in a sylvatic lifecycle involving wild canids in Québec [7, 10, 39]. Only *E*. *multilocularis* was tested at the species level using the RT-PCR test. Based on previous studies, we suspect that *E*. *canadensis* and *E*. *multilocularis* are the two species composing the *Echinococcus* spp. in Canada. In Alberta (Canada), there was evidence of co-infection with *E*. *canadensis* and *E*. *multilocularis* in the intestinal content of 27% of the 40 coyotes and red foxes tested with qPCR [27]. As genotyping was not performed in our study and considering that co-infections are common, we could not determine if prevalence of *E*. *multilocularis* is higher than *E*. *canadensis* based on RT-PCR results.

*Echinococcus* spp. was detected in all administrative regions sampled, except for the Capitale-Nationale and the Centre-du-Québec (Fig 1). This is particularly surprising for the Centre-du-Québec region, given it is largely covered by agricultural fields [42] that provide environmental conditions favorable for coyotes, and coyote captures are normally numerous in this area [43, 44]. Although we found the highest prevalence of *E*. *multilocularis* in Montérégie (14.67%), it remains lower than what was observed in Alberta (25.3% in coyotes in the Calgary and Edmonton area [31] and Ontario (23% in coyotes and foxes [7]. One of the highest prevalence observed in Canada was in urban Edmonton (Alberta) where 23 coyotes were detected with the parasite, resulting in an estimated prevalence of 65.2% in 2016–2017 [45], while the lowest prevalence of *E*. *multilocularis* was found (7.3%, 9/124) in coyotes in periurban and urban Winnipeg (Manitoba) in 2018 [22].

We collected wild canids over a large territory in Québec and gathered an important sample size, which increased our ability to detect the parasite. In most administrative regions, the number of collected carcasses exceeded the target of 50. However, a lower sample size was obtained in the Capitale-Nationale (41), the Laurentides (44), Montréal (5) and Laval (0), caused by a limited number of trappers who expressed their interest to the research team or a less productive trapping season for some trappers. Thus, as sample size was calculated to detect at least one positive case assuming a prevalence of at least 6%, the absence of detection of RT-PCR-positive samples does not confidently support the absence of the parasite.

As coyotes and red foxes are definitive hosts for *Echinococcus* spp., they normally do not show clinical signs of infection. Thus, the presence of the parasite in the intestines of definitive hosts should not influence their probability of being captured by trappers [46], which supports the representativeness of collected carcasses. In addition, in Québec, the same baits and trapping systems are used for these species, which should favor comparability in the prevalence estimates between coyotes and red foxes. However, the regional population of coyotes and red foxes in Québec remains unknown. Consequently, the validity of the overall prevalence estimates outside high-risk areas relies upon either the hypothesis of a relatively homogeneous risk of infection across the territory.

We found two areas at higher risk of infection. The first area was centered in Montérégie and corresponded to the clusters A, C, and D. We suspect that these clusters detected the same underlying high-risk area for *E*. *multilocularis* infection, mostly driven by coyotes (Table 2). All but one of the canids included in these three clusters were positive for *E*. *multilocularis*. This high-risk area could result from a movement of coyotes from the northern states of United States or southern Ontario, areas recognized as endemic for *E*. *multilocularis* [7]. Definitive hosts could also have moved from the west of the country to eastern Canada [47].

The second high-risk area, centered in Bas-St-Laurent, corresponds to cluster B and probably involves *E*. *canadensis*. This high-risk area was only detected when red foxes and coyotes were combined in the analysis, suggesting common drivers for the higher risk of infection in this area for those two animal species. Of the 9 positive cases in the cluster B, 9 were positive for *Echinococcus* spp. and 4 were also positive for *E*. *multilocularis*. The high density of moose recorded in this region (7/10 km^2^), which is probably supported by the presence of a young forest on 43% of the landscape and the absence of natural predator like wolves, suggests that this area could likely sustain *E*. *canadensis* parasitic cycle [43, 48].

Other studies also reported high-risk areas of *Echinococcus* spp. [49, 50], which highlights the need to assess risk over smaller areas [14, 18, 45]. Regional variations in host density and other environmental factors such as weather and land cover, including forest types, road density and human population density, are potential risk factors that could shape the emergence of *Echinococcus* high-risk areas of infection [7, 51, 52].

In our study, *Echinococcus* spp. was detected in both coyotes (5.7%) and red foxes (4.9%), but no difference between species was detected as for *E*. *multilocularis*. In contrast, differences between canid species were observed in a previous study conducted in southern Québec and Maine, with *Echinococcus* spp. detected in coyotes (14%) and wolves (35%), but not in red foxes [10]. Coyotes and red foxes are traditionally sympatric, spatially segregated and competitive species [30, 53]. In an urban environment, it is suspected that coyotes and red foxes are more tolerant to cohabitation [30] which could likely influence the risk of infection. In our study, the prevalence between males and females was not significantly different. Although some studies reported a significantly higher prevalence of infection in males compared with females [31, 54], sex is not considered a significant predictor of infection [45]. Indeed, males and females coyotes have similar behavior and home range size, which may explain our results [40].

We observed a significantly higher risk of *Echinococcus* spp. detection in intestinal content showing *Taenia* spp. eggs in coproscopy. However, only 8 of the 25 positive wild canids for *E*. *multilocularis* in RT-PCR were detected by coproscopy. This low sensitivity of coproscopy is a limitation for use as a screening tool for *Echinococcus* in wild canids. This is consistent with the results of another study where sensitivity for taeniid egg detection was 70% with a limit detection of 10–25 eggs/gram of feces [35]. RT-PCR has the advantages of being a rapid and easy test performed on a large number of fecal samples [55] and it can also detect parasite fragments unlikely to be detected by coproscopy [35].

Wild canids, such as coyotes and foxes, are highly adaptable to urban and suburban environments and can tolerate closeness to humans, which is supported by their increasing presence in many metropolitan areas across North America [29, 56, 57]. A higher abundance of definitive hosts in an urban area rises the risk of environmental contamination and, subsequently exposure at the human-domestic dogs-wildlife interface [58]. However, the transmission pathways from wild canids to domestic dogs have yet to be elucidated. In Alberta (Canada), despite a 25% prevalence of *E*. *multilocularis* in urban coyotes [31], the prevalence in domestic dogs frequenting urban off-leash dog parks was 0.2% (95% CI 0.0–0.7%) [36]. Also, even if human seroprevalence is higher in *E*. *multilocularis* endemic areas, the incidence of human cases does not necessarily correlate with the degree of environmental contamination or prevalence of infection in definitive and intermediate hosts [59]. This could be linked to the long incubation period in humans, which delays case detection. Moreover, in humans, the risk of infection can be influenced by lifestyle, socioeconomic status or environmental conditions such as urban park extent [36], whereas the risk of disease development likely depends on immunity [15, 59, 60]. In Canada, the highest number of human echinococcosis cases were recorded in the provinces of Ontario (178 cases) and Alberta (51 cases) from 2002–2011 [5], whereas only 1 case has yet been detected in Québec [34].

Considering that the clinical sensitivity and specificity of the RT-PCR test we used were not available, the estimation of the true prevalence was not possible in our study. Nevertheless, given the high analytical specificity of two RT-PCR, we consider our estimated prevalence as a likely underestimation of the true risk of infection. False negative PCR results could occur in the presence of low (<10) worm burden, immature worms only, low count (<2) of eggs per gram, or the presence of inhibiting substances in the intestinal content [61–65]. Related to that, in our study, the CT values obtained are highly variable between fecal samples for both *Echinococcus* spp. and *E*. *multilocularis*, suggesting an heterogeneity in the egg concentrations [65–67]. Even if considered rare, false positive RT-PCR results could be observed if a canid ingests a non-infectious but infected rodent [68]. However, this excretion of DNA in stools is short-term (a day or less) compared to an infected canid that excretes intermittently during 2–5 months [55, 68].

## Conclusion

We detected *E*. *multilocularis* in wild canids for the first time in Québec, a province formerly considered free from *E*. *mutlilocularis*. Although the prevalence of *Echinococcus* remains low in wild canids of the current study, the parasite was detected in several regions of Québec, with two areas at higher risk of infection. Diagnosis of an infection could not rely only on coproscopy and must be determined and confirmed with an RT-PCR test. This widespread of the parasite highlights the need for developing targeted prevention programs for domestic dog owners, trappers and other people at risk.

## Supporting information

S1 Fig Distribution of Ct values in RT-PCR-positive canids Echinococcus spp. (n = 36) in coyotes and red foxes from Québec, Canada. (TIFF)

S2 Fig Distribution of Ct values in RT-PCR-positive canids for E. multilocularis (n = 25) in coyotes and red foxes from Québec, Canada. (TIFF)

S3 Fig Distribution of coyotes (n = 423) according to their RT-PCR status for Echinococcus spp. in Québec (Canada), 2020–2021. Numbers identify administrative regions (01 –Bas-St-Laurent, 02 –Saguenay-Lac-St-Jean, 03 –Capitale-Nationale, 04 –Mauricie, 05 –Estrie, 06 –Montréal, 07 –Outaouais, 09 –Côte-Nord, 11 –Gaspésie-Îles-de-la-Madeleine, 12 –Chaudières-Appalaches, 13 –Laval, 14 –Lanaudière, 15 –Laurentides, 16 –Montérégie, 17 –Centre-du-Québec).
(TIF)

S4 Fig Distribution of coyotes (n = 423) according to their RT-PCR status for E. multilocularis in Québec (Canada), 2020–2021. Numbers identify administrative regions (01 –Bas-St-Laurent, 02 –Saguenay-Lac-St-Jean, 03 –Capitale-Nationale, 04 –Mauricie, 05 –Estrie, 06 –Montréal, 07 –Outaouais, 09 –Côte-Nord, 11 –Gaspésie-Îles-de-la-Madeleine, 12 –Chaudières-Appalaches, 13 –Laval, 14 –Lanaudière, 15 –Laurentides, 16 –Montérégie, 17 –Centre-du-Québec).
(TIF)

S5 Fig Distribution of red foxes (n = 284) according to their RT-PCR status for Echinococcus spp. in Québec (Canada), 2020–2021. Numbers identify administrative regions (01 –Bas-St-Laurent, 02 –Saguenay-Lac-St-Jean, 03 –Capitale-Nationale, 04 –Mauricie, 05 –Estrie, 06 –Montréal, 07 –Outaouais, 09 –Côte-Nord, 11 –Gaspésie-Îles-de-la-Madeleine, 12 –Chaudières-Appalaches, 13 –Laval, 14 –Lanaudière, 15 –Laurentides, 16 –Montérégie, 17 –Centre-du-Québec).
(TIF)

S6 Fig Distribution of red foxes (n = 284) according to their RT-PCR status for E. multilocularis in Québec (Canada), 2020–2021. Numbers identify administrative regions (01 –Bas-St-Laurent, 02 –Saguenay-Lac-St-Jean, 03 –Capitale-Nationale, 04 –Mauricie, 05 –Estrie, 06 –Montréal, 07 –Outaouais, 09 –Côte-Nord, 11 –Gaspésie-Îles-de-la-Madeleine, 12 –Chaudières-Appalaches, 13 –Laval, 14 –Lanaudière, 15 –Laurentides, 16 –Montérégie, 17 –Centre-du-Québec).
(TIF)

S1 Table Prevalence with 95% exact CI of RT-PCR-positive Echinococcus spp. and E. multilocularis by administrative region in 423 coyotes in Québec, Canada (2020–2021). (DOCX)

S2 Table Prevalence with 95% exact CI of RT-PCR-positive Echinococcus spp. and E. multilocularis by administrative region in 284 red foxes in Québec, Canada (2020–2021). (DOCX)
