# Supplementary figures and images for: Prevalence and geographic distribution of Echinococcus genus in wild canids in southern Québec, Canada
Source: PLoS One. 2024 Jul 15;19(7):e0306600. doi: 10.1371/journal.pone.0306600 (PMC11249250; doi:10.1371/journal.pone.0306600)

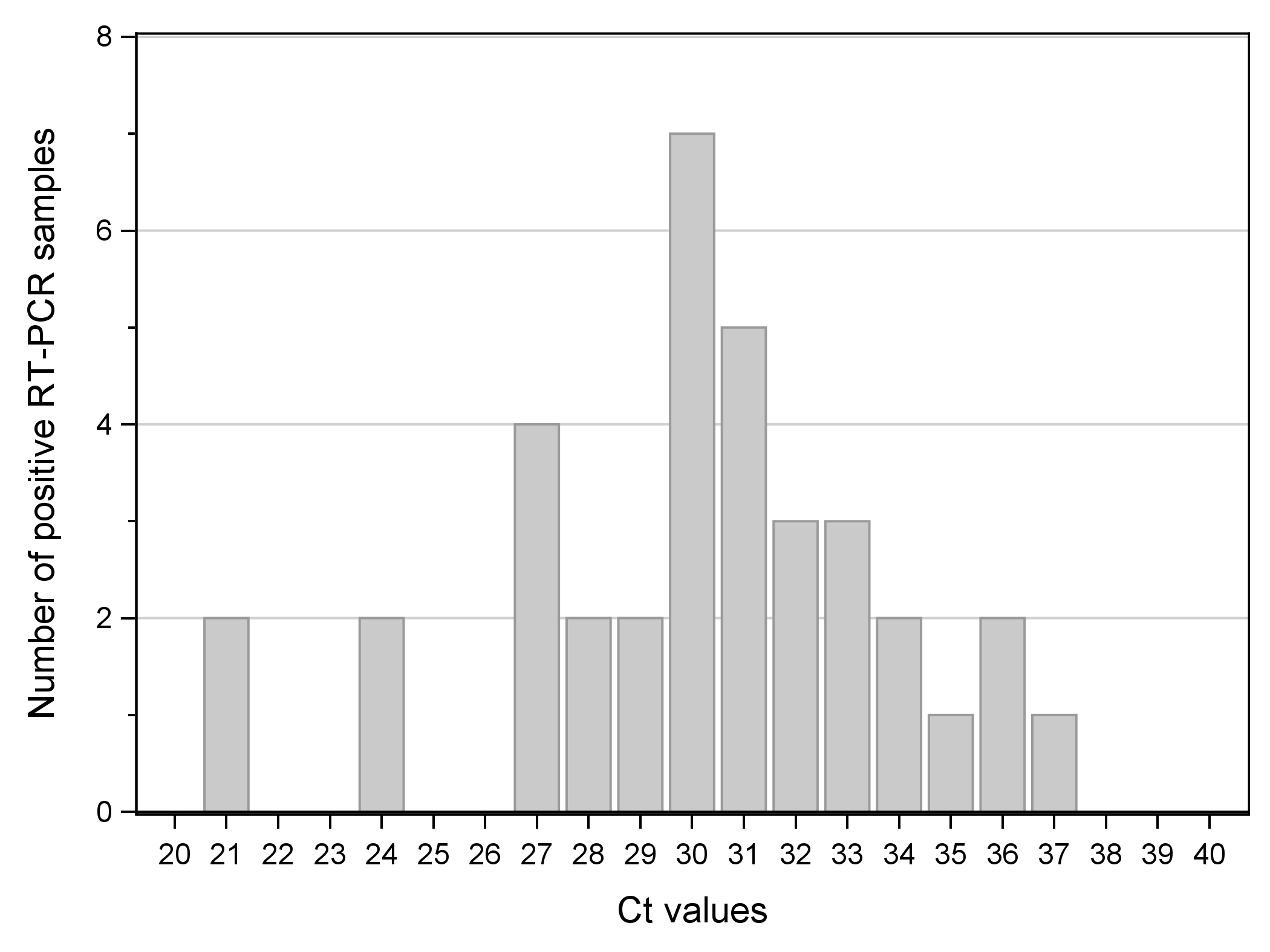

Supplement: S1 Fig — (TIFF) [file pone.0306600.s001.tiff]

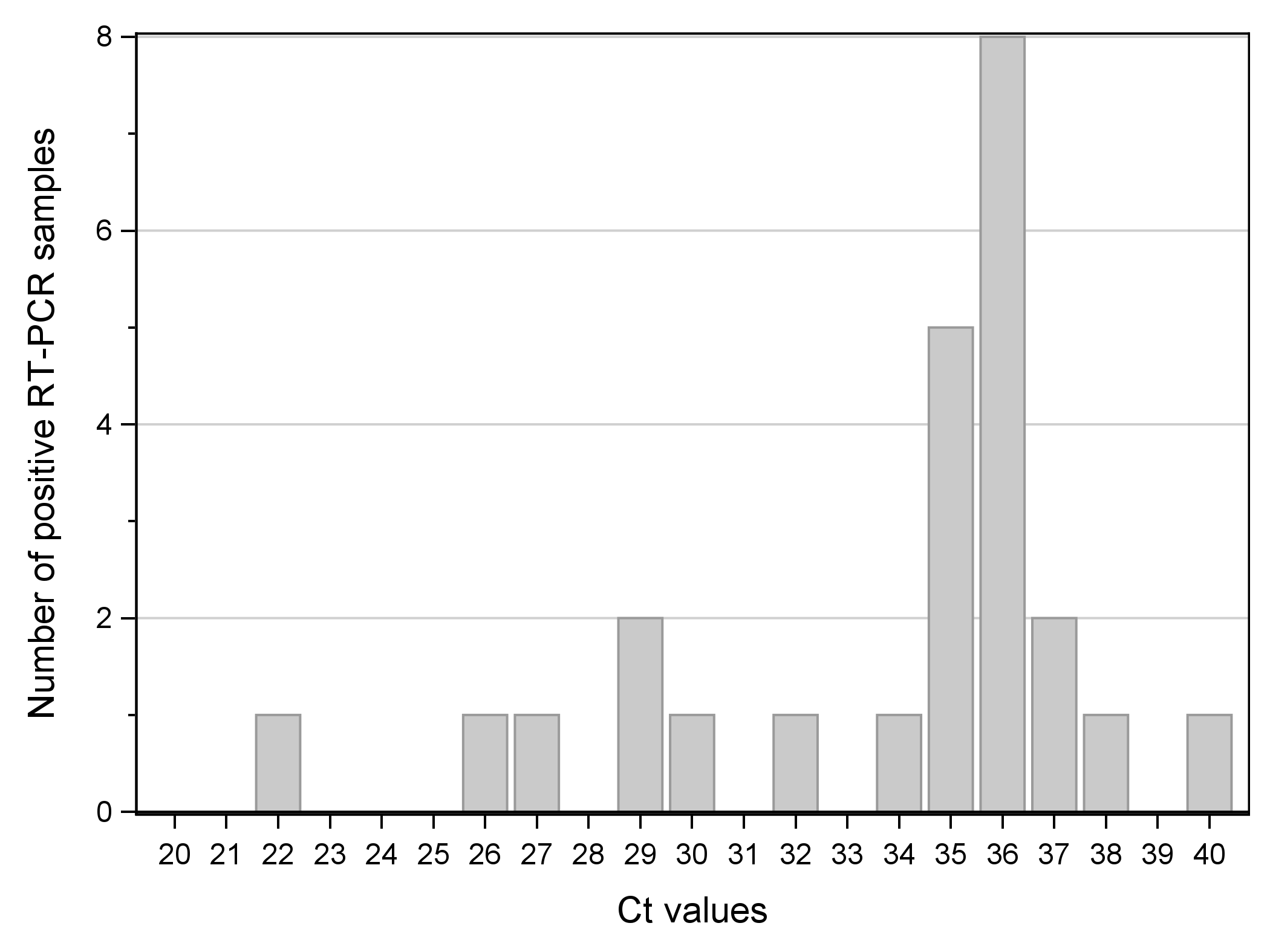

Supplement: S2 Fig — (TIFF) [file pone.0306600.s002.tiff]

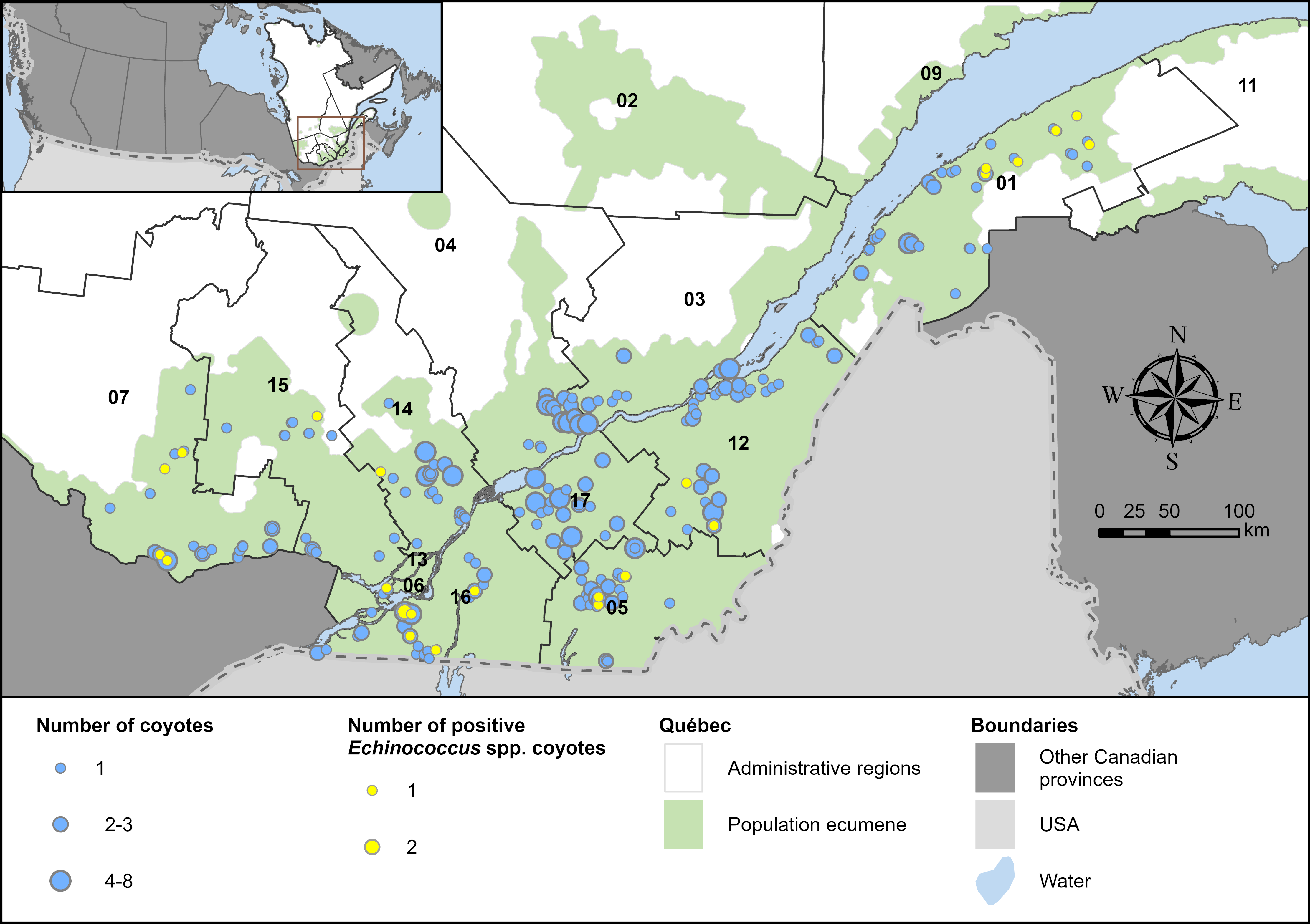

Supplement: S3 Fig — Numbers identify administrative regions (01 –Bas-St-Laurent, 02 –Saguenay-Lac-St-Jean, 03 –Capitale-Nationale, 04 –Mauricie, 05 –Estrie, 06 –Montréal, 07 –Outaouais, 09 –Côte-Nord, 11 –Gaspésie-Îles-de-la-Madeleine, 12 –Chaudières-Appalaches, 13 –Laval, 14 –Lanaudière, 15 –Laurentides, 16 –Montérégie, 17 –Centre-du-Québec). (TIF) [file pone.0306600.s003.tif]

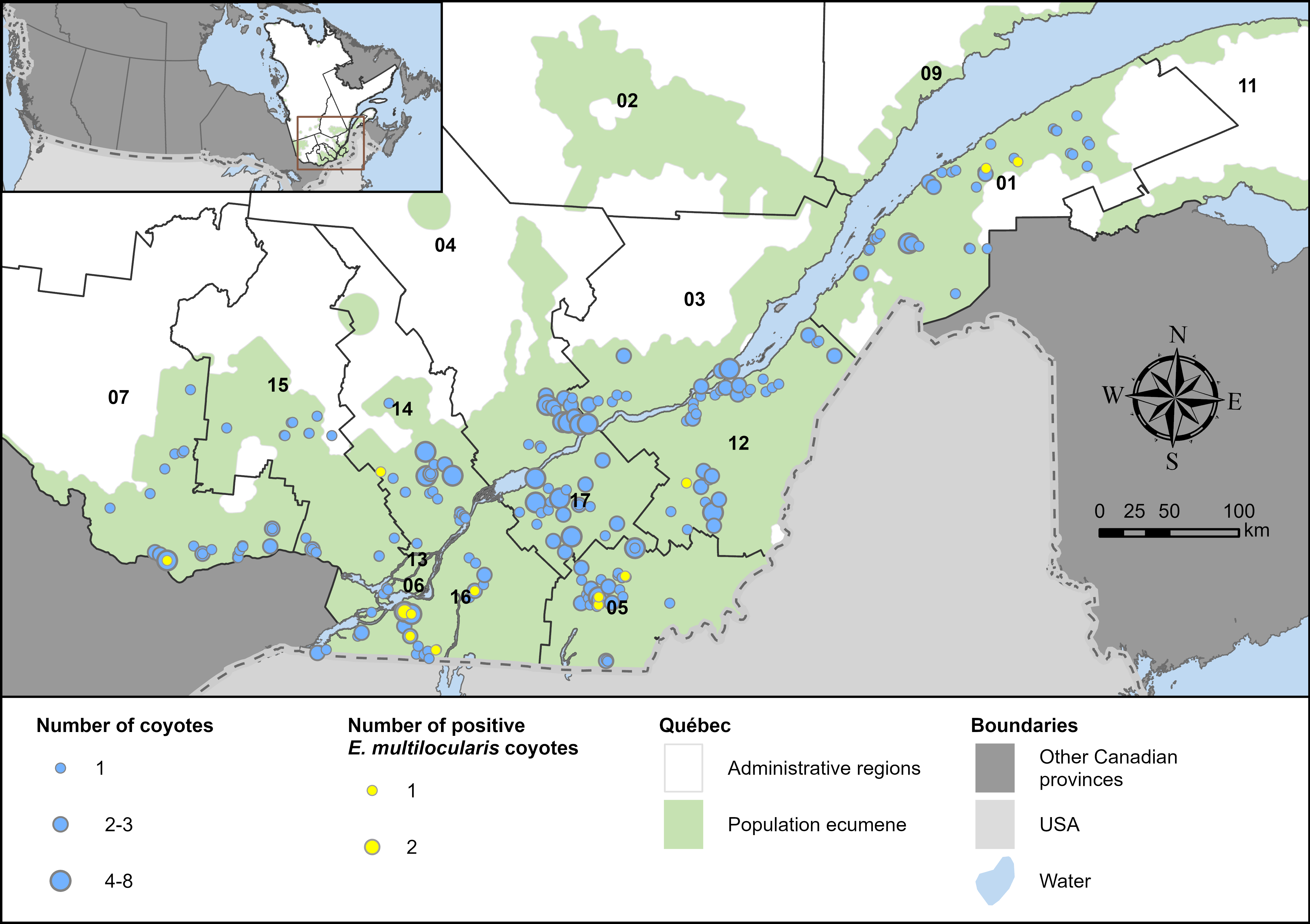

Supplement: S4 Fig — Numbers identify administrative regions (01 –Bas-St-Laurent, 02 –Saguenay-Lac-St-Jean, 03 –Capitale-Nationale, 04 –Mauricie, 05 –Estrie, 06 –Montréal, 07 –Outaouais, 09 –Côte-Nord, 11 –Gaspésie-Îles-de-la-Madeleine, 12 –Chaudières-Appalaches, 13 –Laval, 14 –Lanaudière, 15 –Laurentides, 16 –Montérégie, 17 –Centre-du-Québec). (TIF) [file pone.0306600.s004.tif]

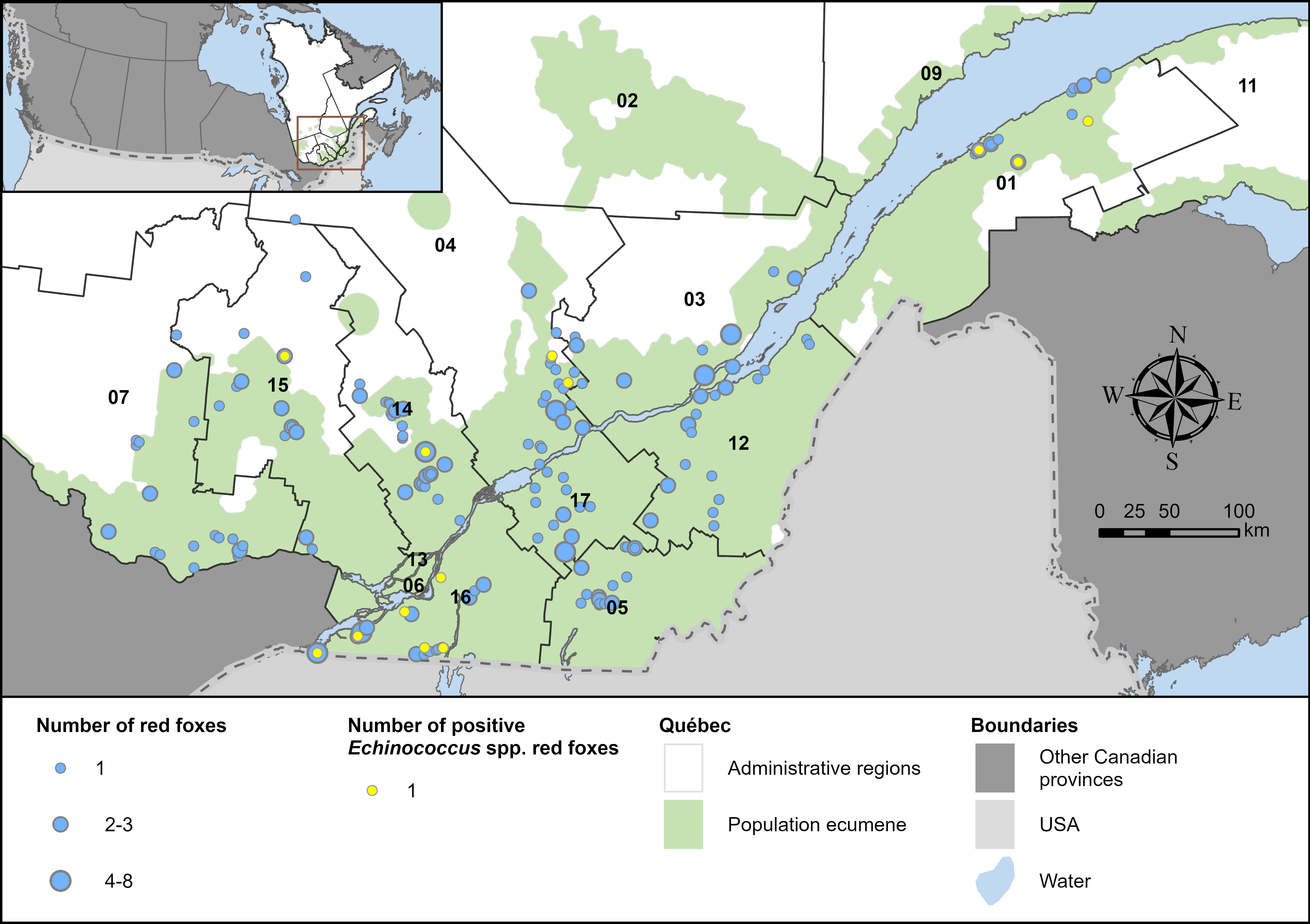

Supplement: S5 Fig — Numbers identify administrative regions (01 –Bas-St-Laurent, 02 –Saguenay-Lac-St-Jean, 03 –Capitale-Nationale, 04 –Mauricie, 05 –Estrie, 06 –Montréal, 07 –Outaouais, 09 –Côte-Nord, 11 –Gaspésie-Îles-de-la-Madeleine, 12 –Chaudières-Appalaches, 13 –Laval, 14 –Lanaudière, 15 –Laurentides, 16 –Montérégie, 17 –Centre-du-Québec). (TIF) [file pone.0306600.s005.tif]

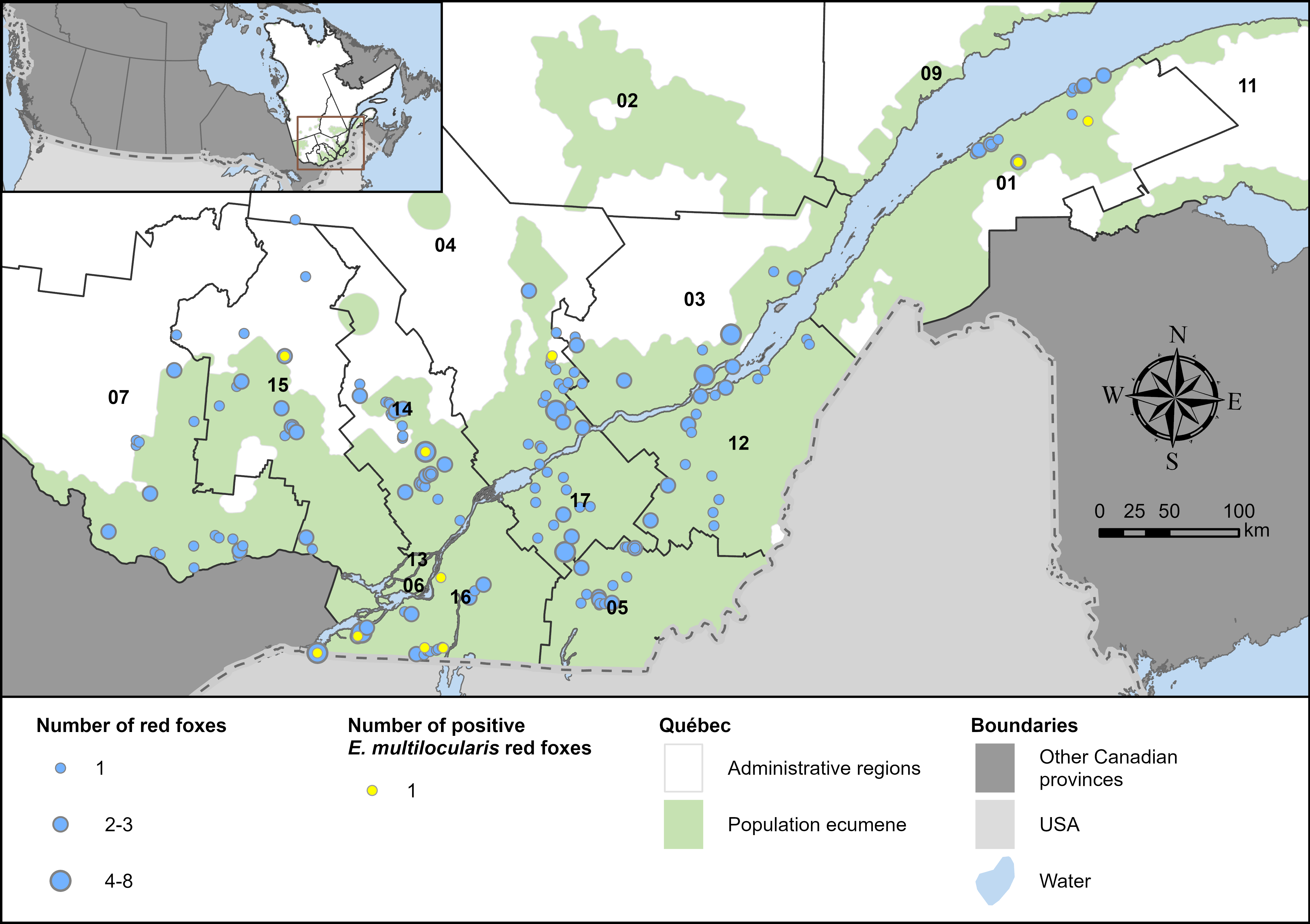

Supplement: S6 Fig — Numbers identify administrative regions (01 –Bas-St-Laurent, 02 –Saguenay-Lac-St-Jean, 03 –Capitale-Nationale, 04 –Mauricie, 05 –Estrie, 06 –Montréal, 07 –Outaouais, 09 –Côte-Nord, 11 –Gaspésie-Îles-de-la-Madeleine, 12 –Chaudières-Appalaches, 13 –Laval, 14 –Lanaudière, 15 –Laurentides, 16 –Montérégie, 17 –Centre-du-Québec). (TIF) [file pone.0306600.s006.tif]
