# Supplementary material for: Prevalence and geographic distribution of Echinococcus genus in wild canids in southern Québec, Canada
Source: PLoS One. 2024 Jul 15;19(7):e0306600. doi: 10.1371/journal.pone.0306600 (PMC11249250; doi:10.1371/journal.pone.0306600)
Supplement: S1 Table — (DOCX) [file pone.0306600.s007.docx]

**S1 Table – Prevalence with 95% exact CI of RT-PCR-positive *Echinococcus*spp. and *E. multilocularis* by administrative region in 423 coyotes in Québec, Canada (2020-2021).**

| Administrative region | Number of coyotes | *Echinococcus* spp. | | *E. multilocularis* | |
| --- | --- | --- | --- | --- | --- |
|  |  | % of positives | 95% exact CI | % of positives | 95% exact CI |
| Bas-St-Laurent | 51 | 11.8 | 4.4 – 23.9 | 3.9 | 0.5 – 13.5 |
| Capitale-Nationale | 20 | 0 | 0.0 – 16.8 | 0 | 0.0 – 16.8 |
| Centre-du-Québec | 50 | 0 | 0.0 – 7.1 | 0 | 0.0 – 7.1 |
| Chaudière- Appalaches | 53 | 3.8 | 0.5 – 13.0 | 1.9 | 0.1 – 10.1 |
| Estrie | 53 | 5.7 | 1.2 – 15.7 | 5.7 | 1.2 – 15.7 |
| Lanaudière | 44 | 2.3 | 0.1 – 12.0 | 2.3 | 0.1 – 12.0 |
| Laurentides | 21 | 4.7 | 0.1 – 23.8 | 0 | 0.0 – 16.1 |
| Laval | 0 | – | – | – | – |
| Mauricie | 47 | 0 | 0.0 – 7.6 | 0 | 0.0 – 7.6 |
| Montréal | 5 | 20.0 | 0.5 – 71.6 | 0 | 0.0 – 52.2 |
| Montérégie | 36 | 16.7 | 6.4 – 32.8 | 16.7 | 6.4 – 32.8 |
| Outaouais | 43 | 9.3 | 2.6 – 22.1 | 2.3 | 0.1 – 12.3 |
