# Supplementary material for: Prevalence and geographic distribution of Echinococcus genus in wild canids in southern Québec, Canada
Source: PLoS One. 2024 Jul 15;19(7):e0306600. doi: 10.1371/journal.pone.0306600 (PMC11249250; doi:10.1371/journal.pone.0306600)
Supplement: S2 Table — (DOCX) [file pone.0306600.s008.docx]

**S2 Table - Prevalence with 95% exact CI of RT-PCR-positive *Echinococcus*spp. and *E. multilocularis* by administrative region in 284 red foxes in Québec, Canada (2020-2021).**

| Administrative region | Number of red foxes | *Echinococcus* spp. | | *E. multilocularis* | |
| --- | --- | --- | --- | --- | --- |
|  |  | % of positives | 95% exact CI | % of positives | 95% exact CI |
| Bas-St-Laurent | 25 | 12.0 | 2.6 – 31.2 | 8.0 | 1.0 – 26.0 |
| Capitale-Nationale | 21 | 0 | 0.0 – 16.1 | 0 | 0.0 – 16.1 |
| Centre-du-Québec | 22 | 0 | 0.0 – 15.4 | 0 | 0.0 – 15.4 |
| Chaudière- Appalaches | 24 | 0 | 0.0 – 14.3 | 0 | 0.0 – 14.3 |
| Estrie | 28 | 0 | 0.0 – 12.3 | 0 | 0.0 – 12.3 |
| Lanaudière | 44 | 2.3 | 0.1 – 12.0 | 2.3 | 0.1 – 12.0 |
| Laurentides | 23 | 4.4 | 0.1 – 22.0 | 4.4 | 0.1 – 22.0 |
| Laval | 0 | – | – | – | – |
| Mauricie | 34 | 8.8 | 1.9 – 23.7 | 5.9 | 0.7 – 19.7 |
| Montréal | 0 | – | – | – | – |
| Montérégie | 39 | 15.4 | 5.9 – 30.5 | 12.8 | 4.3 – 27.4 |
| Outaouais | 24 | 0 | 0.0 – 14.3 | 0 | 0.0 – 14.3 |
